# Discovery of new biomarkers for malignant mesothelioma

**DOI:** 10.1007/s13665-015-0106-8

**Published:** 2015-01-31

**Authors:** Jenette Creaney, Ian M. Dick, Bruce W. S. Robinson

**Affiliations:** 1 National Centre for Asbestos Related Diseases, School of Medicine and Pharmacology, University of Western Australia, 35 Stirling Hwy, Crawley, 6009 Australia; 2 Australian Mesothelioma Tissue Bank, Sir Charles Gairdner Hospital, Verdun Ave, Nedlands, 6009 Western Australia Australia

**Keywords:** Mesothelioma, Biomarker, Mesothelin

## Abstract

Malignant mesothelioma is an asbestos-induced, aggressive tumour with limited treatment options and very poor outcome. Currently, there are no tumour biomarkers in widespread clinical use for this disease. Soluble mesothelin is the most intensively investigated mesothelioma biomarker and has been approved by the US FDA primarily as a tool for monitoring patient response and progression. Mesothelin is elevated in the blood and effusions of patients with mesothelioma, and is rarely elevated in people with benign disease with normal renal function. However, the sensitivity of mesothelin limits its use as a stand-alone tool for the screening of the asymptomatic asbestos-exposed population—one of the primary aims of mesothelioma biomarker studies. Thus, there is an intense research effort focused on the identification of new and/or novel biomarkers for mesothelioma. Some of the challenges associated with biomarker discovery in mesothelioma are discussed.

## Introduction

Intense interest has focussed on identifying and validating new biomarkers for disease, including for cancer. The first of the so-called traditional circulating blood cancer biomarkers, carcinoembryonic antigen (CEA) and alpha-fetoprotein were identified in the 1960s. Since then, only 13 blood-based and four urine-based cancer biomarkers have received approval for clinical use from the US Food and Drug Administration (FDA), a level of approval which is considered the “gold standard” for clinically useful biomarker discovery [1].

A large number of candidate biomarkers have been reported in the literature. For these new biomarkers to be brought to the clinic, they must offer advantages over existing biomarkers or clinical procedures in terms of improved sensitivity and specificity or prognostic or predictive ability and must fulfil specific clinical needs, as well as being cost-effective [2]. The majority of biomarkers approved by the FDA are approved for use in a monitoring setting. However, biomarkers have been proposed for many different settings of disease management, including diagnosis, prognosis, risk stratification, guiding therapy selection, overall management and population screening. Interests in biomarker discovery not only stem from the potential health benefits but also the huge economic potential following the introduction of a new test into the health care system [3].

## Malignant mesothelioma

For mesothelioma, an asbestos-induced tumour with very poor patient outcome, 5-year survival rates of less than 5 % are routinely reported [4]. Finding a biomarker that could enable early detection of mesothelioma, before symptoms develop, has been a driving focus for researchers. There is evidence that screening can impact on cancer-specific mortality particularly for breast, bowel and cervical cancers with reports of significant decreases in disease-specific mortality following screening programmes [5]. However, for mesothelioma, no effective treatment is available. Proponents of early diagnosis for mesothelioma suggest that a timely diagnosis provides the opportunity to offer treatment to patients at an earlier time when tumours are smaller, localised and more accessible to treatments. There is some limited evidence to support this; intra-cavity immunotherapeutic trials from France showed that if malignant mesothelioma was detected early, the disease could potentially be treated in a less aggressive manner and more successfully [6]. Also, in a non-trial setting, a selected group of patients, those presenting with early stage disease and treated with multimodality therapy (surgery, postoperative chemotherapy with or without radiation therapy), have been reported to have a 46 % 5-year survival [7].

Clearly, differences exist in the requirements for a biomarker intended as a screening tool for a population compared to a biomarker for triaging symptomatic individuals. In individuals already presenting with symptoms, the risks associated with surgical confirmation of a malignant diagnosis would generally be acceptable whereas the rate of false positives in a screening programme would need to be minimised if surgery was to be a confirmatory tool. Thus, a biomarker with very high specificity would be required in such a setting. False positives could be minimised by only screening high risk individuals, and for mesothelioma, this would be those exposed to a moderately high level of asbestos. However, data on asbestos exposure is not well standardised. Many biomarker studies in the literature present patient-reported categorical data (i.e. exposed/unexposed/unknown), as opposed to data derived from lung asbestos fibre burden, job exposure matrixes or correlates with atmospheric asbestos loads.

## Mesothelin

We have been studying mesothelin as a biomarker for malignant mesothelioma for over 12 years. Mesothelin was originally identified as a glycophoshatidylinositol-linked glycoprotein of approximately 40 kDa present on the cell surface of mesothelial cells and some cancer types including ovarian, pancreatic and lung carcinomas; squamous cell carcinomas of the oesophagus and cervix and mesothelioma [8]. In collaboration with Ingegerd and Karl Eric Hellstrom, we identified that a soluble form of mesothelin was present in the blood at high levels in mesothelioma patients [9]. At the time, this protein was termed soluble mesothelin-related peptide (SMRP). Subsequently, it has been found that the detected protein is in the majority a soluble form of the cell surface mesothelin identified by Pastan and colleagues in 1994 that is shed into the circulation [10, 11].

In our original publication, we described elevated levels of mesothelin in the blood of 84 % of a relatively modest total number of mesothelioma patient samples (*n* = 44) using an in-house assay [9]. For comparison, we examined levels of the protein in samples collected in our clinics from patients with other malignant and benign pulmonary diseases, some of whom had been exposed to asbestos, and also from a cohort of younger, female, healthy laboratory volunteers. In these early studies, a positive cut-off value was defined as three standard deviations above the average mesothelin value for the non-asbestos-exposed controls. Using this cut-off, 1 of 22 patients with asbestosis and 1 of 22 patients with idiopathic pulmonary fibrosis, plus 1 of 22 patients with non-small cell lung cancer were mesothelin positive; 157 patients with inflammatory or malignant pulmonary or pleural diseases other than MM were negative. Of the positive patients, one had an elevated creatinine at the time of the sample, a factor since shown to elevate blood mesothelin levels.

In hind-sight, there were a number of issues with the original design and implementation of the 2003 study. Firstly, the cut-off was defined in the same sample set as the sensitivity and specificity was calculated from and also that the control “healthy group” were markedly different from the cases. However, a strength of the study was the relatively large number of clinically relevant controls examined, controls that were collected in the same centre, under the same conditions and in the same time frame as the cases.

The mesothelin assay was advanced for commercial development, and the availability of this standardised assay which was simple and easy to use enabled many different laboratories around the world to independently verify that mesothelin was elevated in the blood of mesothelioma patients. Indeed, a meta-analysis examining the data from 16 studies at eleven different laboratories from 1026 individual mesothelioma patients and 3465 controls of various aetiologies, including healthy individuals, those with benign disease and those with malignancy, concluded that elevated serum mesothelin is a strong predictor for mesothelioma, but that the marker lacks the sensitivity to be used in early diagnosis [12••].

## Biomarker discovery

Various genomic, proteomic, immunomic, imaging and other tools have been used to identify, quantify and characterise novel biomarkers for many different diseases. There are advantages and disadvantages to the various biomarker discovery platforms. Common problems for all platforms include the high false discovery rate and the identification of tens to hundreds of potential biomarkers in discovery phases that need to be validated. There is also a poor rate of conversion of biomarkers identified in discovery projects to clinically validated bioassays that provide favourable health impacts. Several biomarker discovery pipelines have been proposed to maximise the efficiency of translation of biomarker discovery to clinical implementation. The process can be broken into six steps: discovery, validation, verification, assay development, retrospective clinical validation and prospective clinical evaluation and then followed by, the not unchallenging steps, of commercialisation and “deployment into the clinic”. Generally speaking, the number of patient samples analysed increases as the candidate biomarker proceeds down the pipeline [13, 14]. Clearly, the steps in the pipeline can be tailored to specific clinical questions or discovery platforms. Critical for the process is that independent validation of biomarker performance, ideally in multiple centres, is performed; however, the acceptable levels of evidence required depend on many factors, particularly the clinical context the biomarker will eventually be put to.

One particularly challenging question for biomarker discovery is the choice of sample type for use in the discovery step. As blood is likely to be the sample that will be used clinically, it is attractive to use this in the discovery phase. However, human blood is a very complex mixture comprising of over tens of thousands of proteins that span more than ten orders of magnitude in terms of protein abundance from albumin at 50 mg/mL down to some cytokines present at the 5 pg/mL range [15]. In addition, the 22 most abundant proteins, which are unlikely cancer biomarkers, account for 99 % of blood protein content and could mask the identification of low abundant proteins where cancer biomarkers are likely to be discovered. Generally, clinical biomarkers (i.e. mesothelin, PSA, etc) are found in the nanogramme/millilitre range, and it is believed that novel biomarkers will be at this concentration or less. The dynamic range and complexity of the plasma proteome causes extreme difficulties in identifying low abundance markers directly in the blood [16]. Therefore, studies have either performed a prior extraction step to remove these abundant proteins or used various alternative fluids in which it is hoped that the tumour markers would be at higher concentration due to proximity to the tumour tissue, and have reduced complexity. Such samples include tumour tissue, pleural effusion and cell lines. However, such sample choice simply present different inherent complications including tumour tissue heterogeneity, the presence (or absence) of stroma and the effect of changes to the cell induced by culturing.

Another fundamental issue in biomarker discovery projects is the choice of comparator group if a biomarker is planned for diagnosis (or ultimately screening). Normal/healthy controls are generally employed in biomarker discovery studies; however, the average mesothelioma patient is a man in his 70s and this group tends to have various comorbidities. The best controls for a diagnostic study would be those patients presenting with symptoms indicative of the disease under study. For screening, a non-symptomatic but otherwise matched population is required. Another useful control for screening studies is longitudinal samples from the same individual.

Further influencing the choice of “normal” control group used for comparative purposes in biomarker discovery studies is the biospecimen type used (i.e. blood, tumour, proximal fluid or cell lines). For example, normal pleural fluid is difficult to ethically obtain; non-malignant (normal) cells in culture generally have significantly different proliferation rates than cancerous cell lines, and the use of immortalised, for example viral transformed cell lines, is particularly artificial.

In a simple case-control comparison, the demonstration of a statistically significant difference in the mean level of the biomarker between groups is not necessarily sufficient to indicate that a biomarker would be clinically useful. Indeed, in some cases, a *t* test *p* < 0.05 is simply reflective of low variability in the control group. Recently, it was elegantly described how the variability of biomarker levels as well as quantitative difference in means between groups gives a better predictor of potential biomarker clinical value. After accounting for such differences and the proportion of cases expressing a given biomarker, Skates and colleagues estimated, for a given discovery set sample size, the probability of validating a given biomarker in the verification stage [17••]. This approach highlights that more factors than the results of a Student’s *t* test need to be accounted for in biomarker discovery studies and that many “true” biomarkers do not necessarily meet the demands of the clinic.

A commonly recognised problem in biomarker studies is that the original study shows great promise but subsequent studies do not have such strong results. Possible reasons for the lack of success in identifying new biomarkers can occur in each of the steps in the discovery pipeline including problems and biases related to experimental design, data analysis, sample collection, processing and/or storage [18, 19]. Furthermore, problems arise from the high variability and the lack of replication performed in some discovery platforms which result in a high rate of false positive candidates. Lack of standardisation in discovery platforms is being addressed by the National Cancer Institute’s Clinical Proteomic Technology Assessment for Cancer Network [20–22]. Filtering of false positive candidates identified in the discovery stage can also be achieved through the use of orthologous techniques in the secondary validation and verification stages using assays with purportedly greater assay precision (such as antibody-based techniques), though some of the problems associated with sampling can be carried over to this step. Another factor that is becoming clearer now is the accuracy of commercially available “research-only” ELISA kits. In our own studies, we have found significant discrepancy between two different ELISAs for the mesothelioma biomarker, megakaryocyte potentiating factor (MPF), whereby in matching samples one assay resulted in a sensitivity of 29 % and the other 52 % at a specificity of 95 % [23, 24]. MPF is the 30-kDa N-terminal protein product of the *MSLN* gene [25, 26]. Using robust assays, MPF conveys equivalent diagnostic information to the mesothelin biomarker in distinguishing mesothelioma from other diseases [23, 27••]. Whilst MPF should be considered an acceptable biomarker for mesothelioma, it is unlikely to be able to contribute anything additional to patient outcomes than the mesothelin biomarker.

## Biomarker discovery in mesothelioma

A review of the literature finds over twenty soluble biomarkers reported for mesothelioma which were identified on a variety of discovery platforms: genomic, proteomic and immunomic. Three recent examples which reported very high diagnostic accuracy in a case-control setting include a panel of 13 biomarkers discovered using SOMAmer proteomic technology [28], fibulin-3 discovered following mRNA expression studies [29] and an autoantibody biomarker panel [30]. Few of these reported mesothelioma biomarkers have yet been independently validated or translated to the clinic and the reasons for this vary.

The SOMAmer panel was identified using aptamer technologies and was reported to have excellent diagnostic accuracy with an area under the receiver operator characteristic (AUC) curve value of 0.99 ± 0.01 in the discovery set and 0.95 ± 0.04 in the validation set of samples for distinguishing mesothelioma cases from asbestos-exposed controls. The 13 component proteins were mostly associated with inflammatory and proliferative functions [28]. To date, however, there have been no follow-up publications. Given the specialised nature of the aptamer platform used for this study, it is unlikely that the panel could be independently validated although with a significant commitment of time and resources, it may be possible to validate the candidates individually using ELISA-based assays.

Several potential mesothelioma candidate biomarkers have been identified principally as differentially expressed molecules following mRNA microarray studies [31–34]. However, in general, there was little consistency in the lists of candidates identified in different studies and of the candidates identified; few have been subject to follow-up biomarker studies. One of the potential candidates that has been examined as a blood-based marker is osteopontin, which was reported to have an AUC of 0.9 (95 % CI 0.82–0.95) for distinguishing mesothelioma stage I cases from asbestos-exposed controls [35]. However, these results were not replicated in independent cohort studies which found that osteopontin had a low specificity for mesothelioma [24, 36], although there were several problems associated with the sample type and sample stability that influenced these findings.

Another potential candidate biomarker identified following mRNA expression studies was fibulin-3, a secreted glycoprotein. Fibulin-3 mRNA was found to be on average significantly overexpressed by 7.36-fold from 37 surgical mesothelioma tumours compared with matched normal peritoneum [31]. In a follow-up study to examine fibulin-3 as a fluid-based biomarker, an initial impressive diagnostic accuracy for mesothelioma was reported; plasma fibulin-3 had a sensitivity of over 96 % at a specificity of 95 %, and pleural effusion fibulin-3 a 84 % sensitivity at a 93 % specificity using a commercial ELISA for the protein (USCN Life Science) [29]. In subsequent sample sets, however, sensitivity was noticeably less, estimated to be 40 % in Canadian samples [29] and 22 % in Australian samples [37] at a 95 % specificity. This is another example of a biomarker that showed great promise in early studies but, as is common with biomarkers, has not subsequently been proven to be useful.

For mesothelioma, as in other disease settings, the vast majority of published biomarker discovery papers report the discovery of a marker with expression levels significantly higher in cases relative to controls. That is statistical significance is the favoured study end-point rather than clinical or practical relevance. Whilst there have been suggestions that biomarker studies not be published until independent validation in external samples is performed, preferably in a clinical setting, we feel this is an unrealistic expectation given the push not only of researchers, but research institutes, universities, the media and even funding bodies for ground-breaking research with a direct clinical impact to be rapidly published. How to reconcile these two aims is not clear. Also, in many instances, if preliminary biomarker evidence remains unpublished due to imperfect validation, then other researchers with the appropriate resources to investigate these biomarkers with their own clinical samples may remain unaware of the potential of the biomarker, and a validation opportunity consequently lost. It is imperative that all suitable studies for a biomarker are reported and subsequently evaluated in a meta-analysis before the final utility of a biomarker can be decided.

## Would we have found mesothelin today using a discovery platform?

Mesothelin was identified as a biomarker for mesothelioma primarily based upon existing biological understanding of the tumour. An interesting question however is, if we were to examine some of the data available from high-throughput screening platforms being used in the untargeted search for novel mesothelioma biomarkers, would we have found mesothelin?

**Fig. 1:**
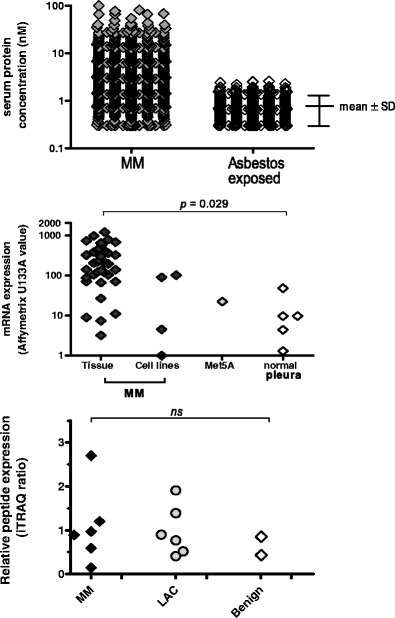
Mesothelin expression. **a** Summary data of serum mesothelin concentrations determined by MESOMARK ELISA in mesothelioma patients (*MM*) and people with asbestos exposure (and normal kidney function) compiled from data collected in our own laboratory over 10 years. **b** Messenger RNA expression data as determined by Affymetrix expression arrays in mesothelioma (*MM*) tissue and cell lines, relative to expression in the SV40-transformed immortalised Met5A mesothelial cell line and in normal pleural tissue as reported by Gordon et al. [34]. **c** Relative mesothelin derived peptide expression as determined by quantitative mass spectrometry-based proteomics in mesothelioma (*MM*), lung adenocarcinoma (*LAC*) and benign pleural effusions as reported by Mundt et al. [40]

The probability of mesothelin being identified in an unbiased biomarker study can be estimated using the assumptions described by Skates and colleagues [17••]. The available data for this purpose for mesothelin as a blood biomarker is that approximately 50 % of mesothelioma cases express the marker at diagnosis, that there is relatively limited variability of mesothelin levels in control samples and there are four or more standard deviations of the mean difference between the mean mesothelin levels in controls relative to cases (Fig. 1a). Using these assumptions, a biomarker discovery experiment limited to ten case and control samples (because of either cost constraints or availability of “normal” tissue) and with 100 samples available for the verification stage, would have approximately a 50 % probability of identifying mesothelin.

Indeed examining the publically available mRNA expression data [38] (GEO accession GSE2549), mesothelin was clearly expressed in mesothelioma surgical specimens whilst not being measurable in four out of five normal pleural tissue samples analysed (Fig. 1b). Although the focus of the Gordon manuscript was not to identify biomarkers but rather improve understanding of mesothelioma tumourgenesis and pathobiology, in supplementary data they show that mesothelin was overexpressed approximately 14-fold in tumours relative to normal lung and pleural tissue (*p* < 0.005). However, examining our own unpublished dataset of mRNA expression in mesothelioma cell lines and normal mesothelial cell cultures, we found no difference in the levels of mesothelin mRNA between the groups. This finding possibly reflects the observation that mesothelin is one of several molecules whose expression is downregulated in tissue culture [39].

Examining publically available quantitative mass spectrometry-based proteomic data, mesothelin was found to be present in the proteome of mesothelioma effusions (Fig. 1c) but was not identified as a candidate biomarker following analysis comparing proteins differentially expressed between mesothelioma and lung cancer [40] (ProteomeXchange identifier PXD000531). In our own unpublished proteomic dataset, mesothelin was only shown to be differentially expressed in one of three experiments.

Thus, whether or not mesothelin would have appeared on any list of candidate biomarkers following an unbiased analysis on a high throughput discovery platform would have been dependent, as expected, upon the biospecimen used (either tumour tissue, cell lines or effusions) as well as the comparator groups evaluated.

## Conclusion

At present, the only example of a FDA-approved biomarker that was identified through a high-throughput screening biomarker discovery strategy is the OVA1® (Vermillion, USA) assay [41•]. Of note, OVA1 is not approved for use in a diagnostic setting but in a specific clinical setting of triaging women with pelvic masses to specific centres for surgical intervention. The OVA1 test consists of four proteins identified by SELDI-TOF mass spectrometry plus CA125, a well-established ovarian cancer biomarker. The panel of markers has a greater specificity than CA125 used alone. We hope that a similar approach, using a panel of biomarkers, might be successful for mesothelioma. Despite a large number of biomarkers being reported for mesothelioma in the literature, mesothelin remains the single-best blood-based biomarker for the cancer and is considered to be the “gold standard” against which new biomarkers need to be judged and remains the only one with FDA approval.
